# Complexity and interplay of faced adversities and perceived health and well-being in highly vulnerable pregnant women—the Mothers of Rotterdam program

**DOI:** 10.1186/s12889-023-14975-7

**Published:** 2023-01-06

**Authors:** L. C. M. Bertens, K. S. C. Mohabier, M. van der Hulst, D. S. E. Broekharst, H. Ismaili M’hamdi, A. Burdorf, R. Kok, J. P. de Graaf, E. A. P. Steegers

**Affiliations:** 1 Department of Obstetrics and Gynaecology, Erasmus University Medical Centre Rotterdam, P.O. Box 2040, 3000 CA Rotterdam, The Netherlands; 2 Department of Medical Ethics and Philosophy of Medicine, Erasmus University Medical Centre Rotterdam, Rotterdam, The Netherlands; 3 Department of Public Health, Erasmus University Medical Centre Rotterdam, Rotterdam, The Netherlands; 4 Erasmus School of Social and Behavioural Sciences Clinical, Child and Family Studies, Erasmus University, Rotterdam, The Netherlands

**Keywords:** Vulnerable populations, Pregnancy, Socioeconomic disadvantage, Adversity, Health, Well-being

## Abstract

**Background:**

Living in socially disadvantaged circumstances has a widespread impact on one’s physical and mental health. That is why individuals living in this situation are often considered vulnerable. When pregnant, not only the woman’s health is affected, but also that of her (unborn) child. It is well accepted that vulnerable populations experience worse (perinatal) health, however, little is known about the lived adversities and health of these vulnerable individuals.

**Objectives:**

With this article, insights into this group of highly vulnerable pregnant women are provided by describing the adversities these women face and their experienced well-being.

**Methods:**

Highly vulnerable women were recruited when referred to tailored social care during pregnancy. Being highly vulnerable was defined as facing at least three different adversities divided over two or more life-domains. The heat map method was used to assess the interplay between adversities from the different life domains. Demographics and results from the baseline questionnaires on self-sufficiency and perceived health and well-being were presented.

**Results:**

Nine hundred nineteen pregnant women were referred to social care (2016–2020). Overall, women had a median of six adversities, distributed over four life-domains. The heat map revealed a large variety in lived adversities, which originated from two parental clusters, one dominated by financial adversities and the other by a the combination of a broad range of adversities. The perceived health was moderate, and 25–34% experienced moderate to severe levels of depression, anxiety or stress. This did not differ between the two parental clusters.

**Conclusions:**

This study shows that highly vulnerable pregnant women deal with multiple adversities affecting not only their social and economic position but also their health and well-being.

**Supplementary Information:**

The online version contains supplementary material available at 10.1186/s12889-023-14975-7.

## Introduction

There is a vast body of literature linking social disadvantaged circumstances to suboptimal health and well-being of individuals. Low levels of health literacy and risky health behaviour, such as smoking, alcohol consumption and an unhealthy diet, have been identified as important risk factors [1–4]. The disadvantaged circumstances themselves are also important factors in explaining health inequalities. For this reason, people living in such circumstances are often considered as vulnerable populations. Prolonged exposure to stress as a result of the disadvantaged circumstances is believed to be an important mechanism in this association [5, 6]. Together, these circumstances create a negative cycle where socioeconomic circumstances influence health and well-being, which in turn affects the economic potential of the individual [7–9]. This is believed to be the main reason for the persistent detrimental effects of poverty [10]. The negative effects are already transferred from one generation to the next during pregnancy, with long lasting effects throughout the entire life course [11–14].

The Mothers of Rotterdam (MoR) Study is a prospective cohort study in highly vulnerable pregnant women, following them and their (unborn) child up to the second birthday of the child. Pregnant women with multiple and interacting adversities on different life domains (e.g., economic, psychosocial and health) were considered to be highly vulnerable. The background and design of the study have been described in detail previously [15]. This study aims to investigate the impact of social care in these highly vulnerable pregnant women on three levels: the social care trajectory, health and well-being of the mother, and health and development of the (unborn child).

Agreement in the literature on the definition of vulnerable populations is limited [16]. The definition of vulnerability used in the Mothers of Rotterdam study is very broad, and this paper aims to investigate which adversities are reported and what the underlying cohesion is. The heat map method, a machine learning technique, is used for this. Moreover, self-sufficiency and the perceived health and well-being of the participating women are investigated. We hypothesized that there was visible cohesion between the different reported adversities, and that the overall self-sufficiency and perceived health and well-being was suboptimal.

## Methods

### Recruitment

Recruitment for the MoR study (January2016—December2020) was open to all pregnant women who resided in Rotterdam, were considered to be highly vulnerable, and were referred to, or applied for, social care [15]. Pregnant women facing a minimum of three adversities over at least two life domains were considered to be highly vulnerable. Application for social care included details of the referring party and a list of adversities indicating the degree of vulnerability of the pregnant woman. Adversities on this list are known to influence self-sufficiency, well-being, and health of the woman or the health of her unborn child. Following application, a social care professional assessed care need and the vulnerability checklist was filled out in the home environment of the pregnant woman. During this visit, the study was explained and eligibility criteria were checked [15]. Social care provision to these women was independent of their participation in the MoR study. Ethical approval for the study was obtained from the Erasmus Medical Centre Ethics Committee (MEC-2016–012).

In the MoR study, different outcomes over three domains are studied; 1) the Care domain, focussing on self-sufficiency and the (social) care process; 2) the Mother domain, focussing on the health and well-being of the mother; and 3) the Child domain, focussing on the health and development of the (unborn) child. The care domain is applicable for all participating women receiving social care, and questionnaire data are collected from the involved social care professionals. The data collected closely resembles routine data used for internal evaluation of provided care, therefore, no informed consent was needed for this part of the study. Participating women did have to explicitly consent to actively contribute to the data collection for the Mother- and Child domains. This resulted in a considerable lower number of participants in these domains. Informed consent was obtained for mother and child separately. Here, insufficient understanding of Dutch, English, Arabic, Polish, Turkish or Spanish, was used as exclusion criterion. This paper describes data from the baseline measures, and since the mothers were pregnant at that time, no data for the children are included.

### Measurements

In this paper we describe the baseline measurements. Two questionnaires were filled out by social care professionals and four by participating pregnant women.

Questionnaires filled out by social care professionals:

- *Vulnerability checklist*: An overview of adversities the pregnant woman faces is made with a checklist containing different adversities within the following domains: pregnancy, residence, finance, occupation, parenting, health, social functioning and safety. This is filled out in the home environment of the pregnant woman by the social care professional during the first visit [15].
- *Self-sufficiency*: This is assessed by the social care professional and measured with the Self-Sufficiency Matrix (SSM) [17]. Self-sufficiency is measured across 11 domains; income, day-time activities, housing, domestic relations, mental health, physical health, addiction, daily life skills, social network, community participation and judiciary. The domains are measured on a 5-point scale with higher scores indicating higher levels of self-sufficiency [18]. For each domain, a score below 4 was considered not self-sufficient.

Questionnaires filled out by the subgroup of pregnant women:

- *Non-verbal IQ*: IQ was measured by an abbreviated nine-item form of the Raven's Standard Progressive Matrices Test [19]. The test provides a nonverbal estimate of fluid intelligence [20]. Lower scores indicated lower fluid intellectual capacity. The scores are categorized into *intellectually impaired* (≤ 35), *below average* (36–46), *average* (47–55), *above average* (56–58), and *intellectually superior* (≥ 59).
- *Smoking*: Data about smoking were collected by asking about the current smoking status of the mother and other persons in the household.
- *Subjective health*: This is measured with the EuroQol Visual Analog Scale (EQ-VAS) [21]. Subjective health was indicated by marking a spot on a vertical line of 10 cm with each millimetre corresponding to 1 point. Lower scores indicate lower subjective health.
- *Depression, anxiety and stress*: This was measured with the 21-item Depression, Anxiety and Stress Scales (DASS-21) [22, 23], containing 21 statements about these constructs and applicability was indicated on a 4-point scale (0 to 3), with higher scores indicating higher applicability of the statement. Scores were categorized per construct into *normal* (0–9 for depression, 0–7 for anxiety, and 0–14 for stress), *mild* (10–13, 8–9, and 15–18), *moderate* (14–20, 10–14, and 19–25), *severe* (21–27, 15–19, and 26–33) and *extremely severe* (≥ 28, ≥ 20, and ≥ 34) [24].

Information on maternal age, address, duration of pregnancy, and referring party were obtained from the application forms. To enable comparison between participants and non-participants, these data were also collected from women who applied for care, but did not participate in the study.

### Statistical analysis

Participating and non-participating women were compared by tabulating mean maternal age, spoken language, residence in a deprived neighbourhood [25], duration of pregnancy at time of referral, and referring party. To investigate the relation between the different adversities scored on the vulnerability checklist, a heat map was created. Heat maps are a method of graphically presenting data where values are depicted by colour, which facilitates visualization and understanding of complex data. An additional benefit of the heat map method is that clustering within the data can be detected and visualized. Here, each participant is represented by a row and the colour on the row depicts the problems faced per life domain. Colour grading is used to visualize the relative number of problems per life domain, with darker colours representing more problems. When all problems within one life domain were present, the score for this domain was set to 10, and when someone scored 2 of the maximum 7 problems, a score of 2.86 was assigned to this domain. Rows in the heat map were sorted based on their difference; meaning that rows that are more different are placed more far apart from each other, and vice versa. This method allows for the detection of hierarchical clustering of the data, which is depicted with a dendrogram next to the heat map.

The overall level of self-sufficiency, according to the social care professionals, was calculated as median score and interquartile range (IQR) over all domains and stratified by participation into the sub-study. Also, the proportion of participants scoring inadequate self-sufficiency were calculated and presented per domain of the questionnaire. Non-verbal IQ, smoking, subjective health and depression, anxiety and stress of the participants contributing to the Mother domain, were compared between the two parental clusters identified with the heat map method. Non-verbal IQ and the depression, anxiety and stress scores were visualized with stacked bar charts showing the distribution of participants over the defined categories of the questionnaires.

The heat map was created using R 4.12 and packages ComplexHeatmap, circlize and seriation. The remainder of the analyses were performed using IBM SPSS Statistics version 28.

## Results

**Fig. 1:**
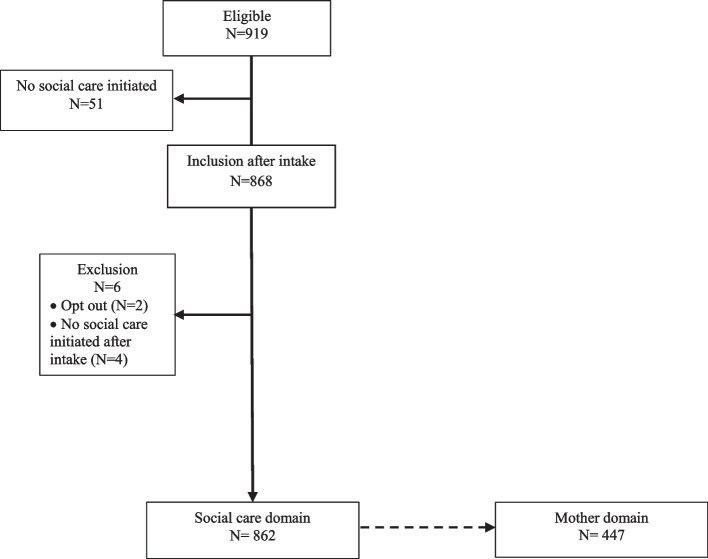
Flow chart of study population

Social care professionals assessed eligibility of all 919 pregnant women applying for social care between January 2016 and December 2020 (Fig. 1). In 51 women, social care was not initiated, resulting in 868 women in the study. Another six women were excluded; in four women targeted social care was not started and two objected to data collection. From the remaining 862 participants, 447 women also actively contributed to the data collection for the Mother- and Child domains.

The mean age was 27.5, 10.7% (*n* = 92) was aged below 20 years, and the vast majority of women spoke Dutch, followed by Arabic and English. Women often lived in a deprived neighbourhood (63.5%), although this was less frequent in the women participating in the sub-study (52.3%). Most women were referred to, or applied for, social care in the second trimester of their pregnancy. Overall, the groups of participating and non-participating pregnant women did not differ from each other (Supplementary table 1).

**Fig. 2:**
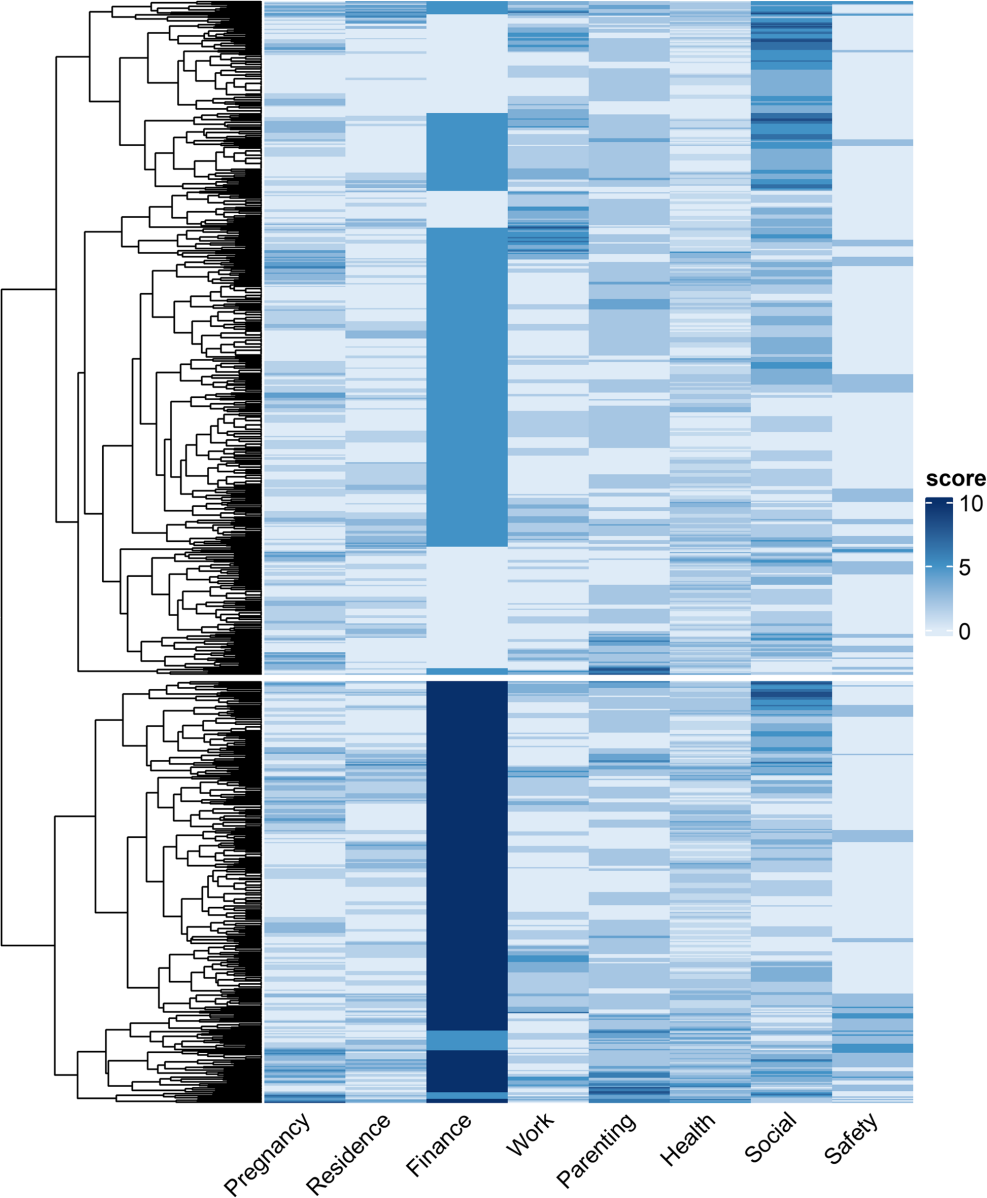
Heat map of the number of adversities scored per life domain for all participants. The colour grading is used to depict the relative number of reported adversities per life domain (x-axis), with darker blue indicating more a problematic situation. The dendrogram on the left of the heat map shows the two parental clusters and all following branches

Findings from the vulnerability checklist are displayed in Table 1. Overall, a median of 6 adversities (IQR 5–8) over 4 (IQR 3–5) life domains were indicated. Adversities in the domains *Social functioning (81.2%), Health (78.1%),* and *Finance* (76.5%) were most frequently scored, and *Parenting (17.9%)* and *Safety and crime (21.7%)* were scored least prevalent. The heat map in Fig. 2 visualises the relative number of adversities per life domain, with darker colours indicating more adversities. The dendrogram left of the heat map visualises the hierarchical clustering of the data. This dendrogram shows that the data consists of a wide range of clusters. Zooming out to the parental branches of the dendrogram, it shows that the data can be divided into two clusters; cluster 1, dominated by more adversities in the finance domain, and cluster 2, where no specific life domain seems to dominate, and which represents the combination of adversities in many life domains.

**Table 1:** Vulnerability checklist

	**All participants**
*Response rate*	*N* = *831 (96.4%)*
**Pregnancy**	476 (57.3)
No prenatal /antenatal care	51 (6.1)
Young or uninformed motherhood/pregnancy	291 (35.0)
Medical problems in unborn child	46 (5.5)
Negative perceptions of the pregnancy	73 (8.8)
Fear of childbirth	145 (17.4)
No reasonable expectations of maternity	37 (4.5)
Unhealthy lifestyle	83 (10.0)
**Residence**	374 (45.0)
Homelessness^a^	213 (25.6)
Imminent eviction	68 (8.2)
No gas/water/electricity	3 (0.4)
Overdue maintenance	55 (6.6)
Unsanitary/unsafe residence	75 (9.0)
Illegal situation (e.g. not registered at address)	98 (11.8)
**Administration and finance**	636 (76.5)
Insufficient income	490 (59.0)
Debt	446 (53.7)
**Work and education**	442 (53.2)
Unemployed	125 (15.0)
No meaningful daily occupation	130 (15.6)
Illiteracy	14 (1.7)
Low education (e.g. < 7 years education)	295 (35.5)
**Parenting**	149 (17.9)
Attachment problems	20 (2.4)
(Expected) parenting problems	87 (10.5)
(Imminent) outplacement of this or previous children	26 (3.1)
Involvement of child protection	33 (4.0)
No basic layette at ≥ 34 weeks of gestation	34 (4.1)
**Health**	649 (78.1)
Physical problems	154 (18.5)
Underweight or overweight	32 (3.9)
Smoking	117 (14.1)
Soft drugs	30 (3.6)
Hard drugs	5 (0.6)
Alcohol	8 (1.0)
Psychiatric problems	154 (18.5)
A lot of stress	531 (63.9)
No health insurance	75 (9.0)
Intellectually disabled	39 (4.7)
**Social functioning**	675 (81.2)
Inadequate social network	405 (48.7)
Single mother	356 (42.8)
Poor communicative skills	123 (14.8)
Language barrier	244 (29.4)
Not self-sufficient	94 (29.4)
Trouble with household chores	51 (6.1)
**Safety and crime**	180 (21.7)
Detention (history) of the mother	27 (3.2)
Detention (history) of the father	37 (4.5)
Illegal immigration status	34 (4.1)
Domestic violence	102 (12.3)

Data are expressed as frequencies (andpercentages) ^a^Homelessness includes sleeping outside, in a homeless shelter or staying at family/ friends

The median self-sufficiency score was 3.91, indicating a suboptimal level (a score below 4) of overall self-sufficiency (Table 2). Participants relatively often scored suboptimal on the domains *Income* (83.5%), *Day-time activities (74.4%),* and *Community involvement* (54.6%). In total, 16 (1.9%) women had a suboptimal level of self-sufficiency on nine or more domains. In contrast, 17 women (2.0%) were considered completely self-sufficient by the social care professional, indicated with self-sufficiency scores of 4 or higher on all 11 domains.

**Table 2:** Baseline assessments – Self-sufficiency

	**All participants**
*Response rate*	*N* = *848 (98.4%)*
**Overall score (median, IQR)**	3.91 (3.55–4.18)
Income	708 (83.5)
Day-time activities	631 (74.4)
Housing	403 (47.5)
Domestic relations	216 (25.5)
Mental health	218 (25.7)
Physical health	86 (10.1)
Addiction	37 (4.4)
Daily life skills	198 (23.3)
Social Network	439 (51.8)
Community involvement	163 (54.6)
Legal	39 (4.6)

Data are expressed as frequencies (and percentages). Self-sufficiency: domain scores < 4, which were considered indicative of a problem

**Fig. 3:**
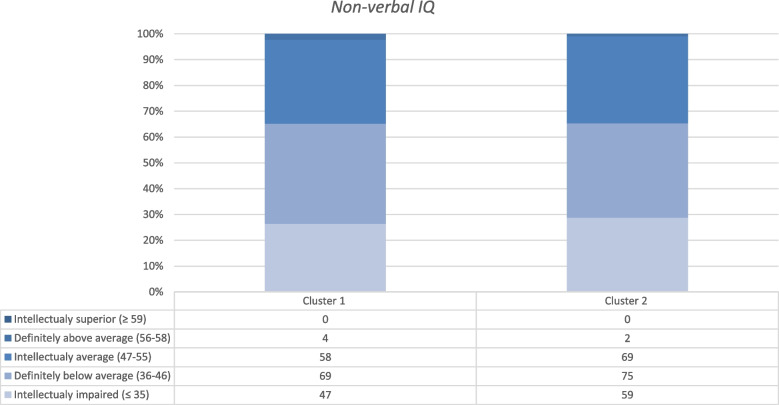
Non-verbal IQ categories stratified by the two parental clusters

**Fig. 4:**
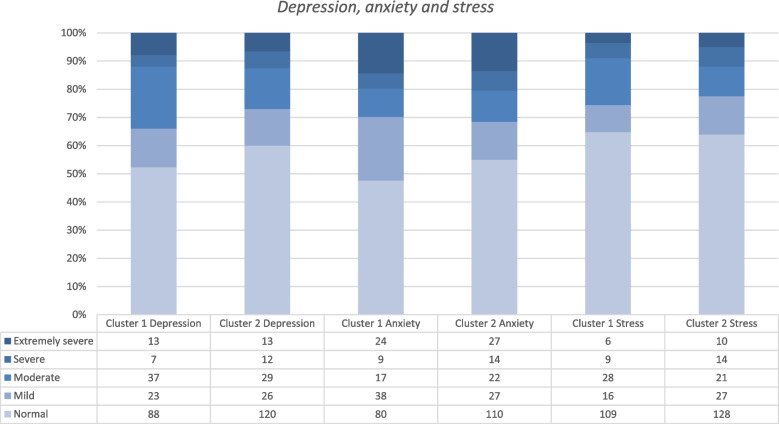
Depression, Anxiety and Stress categories stratified by the two parental clusters

The results from the questionnaires filled out by the individual participants were analysed stratified by the two parental clusters identified by the heat map method (Table 3, Figs. 3 and 4). In cluster 1, which was dominated by high scores on the domain of Finance, participating women reported a lower median subjective health score and were more frequently exposed to active or passive smoking than women assigned to cluster 2 (Table 3).

**Table 3:** Subjective health and smoking status stratified by belonging to one of the two parental clusters

	Cluster 1	Cluster 2
**Subjective health**	*N* = *130*	*N* = *144*
EQ5D-score (median, IQR)	51.0 (38.0–69.3)	54.5 (35.4–75.8)
**Smoking**	*N* = *171*	*N* = *197*
Active smoking	29 (17,0)	27 (13,7)
Passive smoking (i.e. secondhand)	59 (34,5)	58 (29,4)

Data are expressed as median (and interquartile range;IQR) or frequencies (and percentages)

In the majority of women the intellectual capacity was classified as below average (37.4% below average, and 28.0% as intellectually impaired). There were no clear differences present when stratifying the women into clusters 1 or 2 (Fig. 3).

The majority of participating women rated their depression, anxiety and stress levels as normal (50.8%—63.4%). Which is a clear contrast to the percentage of participants reporting high levels of stress in the vulnerability checklist (68.9% in Table 1). Feelings of anxiety were more often reported than the other two constructs, with 20.0% reporting feelings of (extremely) severe anxiety. Feelings of severe or extremely severe depression were reported in 12.6% and feelings of (extremely) severe stress in 11.3% of participants. Again, no notable differences were observed when comparing women belonging to cluster 1 or 2 (Fig. 4).

## Discussion

Our study reveals that highly vulnerable pregnant women experience a wide variety of adversities. This variety shows the multitude of adversities that cover multiple life domains, and thereby influence the complexity of (social) care required for these women. The heat map method used to visualise the reported vulnerabilities, showed a large amount of clusters within the data originating from two parental clusters. One is dominated by multiple problems in the life domain Finance and the other, and largest, cluster is not dominated by a specific life domain and underlines the complex interplay between faced adversities from different life domains. The social care professionals considered the participants least self-sufficient in the domains *Income*, *Day-time activities,* and *Community involvement*. Participating women rated their subjective health as suboptimal, and 34% – 49% experienced mild to extremely severe levels of depression, anxiety and stress. These findings did not really differ between the clusters identified by the heat map.

Many of the adversities the participating pregnant women report are known to influence the health and well-being of the woman and her (unborn) child. Also, demographic characteristics of the women in our study are in line with the literature. Teenage pregnancies and residence in a deprived neighbourhood were more prevalent in our study compared to the national average (11% vs. 1% and 63% vs 9%, respectively) [25, 26], and both are known to adversely influence pregnancy and birth outcomes [27–29]. To our knowledge, this is one of few studies describing a population of highly vulnerable pregnant women in detail. Although the concept ‘vulnerable pregnant women’ is often used to describe a population of pregnant women facing a given adversity, the studied populations differ considerably. Consequently, these different groups of vulnerable pregnant women are not directly comparable, however, they all add to the overall message that this group face many and complex problems and personalized help is required.

When we assessed the connectedness between the different faced adversities, a large number of clusters could be identified. These clusters all originated from one of the two parental clusters. This identification of clusters could be very beneficial for the identification of highly vulnerable women, however the implications of the clustering is not clear. From our cross-sectional data, we are not able to assess whether these clusters need different (social) care or what their prognosis is. Longitudinal data and advanced analytical methods, like machine learning techniques, are needed to study the impact and validity of the identified clusters for (social) care practice and research.

The views of the social care professionals and the participating women converge for the most part. For example, on the vulnerability checklist, 14% of women were active, which is comparable to the self-reported prevalence. Also, the lower intellectual capacity in the population is in line with the amount of individuals with low education. In contrast, the vulnerability checklist showed that 63.9% of women suffered from high levels of stress (Table 1), while the self-reported stress levels in this group where much lower, with 24.5% reporting moderate to extremely severe levels of stress (Table 3). This divergence could be the result of hedonic adaptation, a widespread phenomenon in which people tend to adapt to the state they are in, good or bad, and adjust their baseline utility accordingly [30]. In other words, these women continuously face disadvantaged circumstances and the corresponding effects on their mental health, which then becomes their new normal. When these women are asked to rate their mental health status, they are likely to use different reference levels than individuals not facing the same circumstances.

A major strength of the study is the number of highly vulnerable pregnant women that were included into the study. In most research, highly vulnerable individuals are often underrepresented, and when they are participating it is likely that they represent those better off compared to the non-participating ones. This could also apply to the women in our study, however, a comparison between women who did and did not participate in the study showed no clear differences. We were even able to include women with an illegal status into the study, although the group of women in an illegal situation in need of the care provided by the program is most likely bigger than the group we included. In the Netherlands, everyone is entitled to medical care, irrespective of legal status. By involving obstetric care professionals for the referral to the Mothers of Rotterdam program, the possibility for providing social care to these women with an illegal status was facilitated.

A limitation of the study is that we were not able to test the identified clusters for their longitudinal impact on self-sufficiency and the perceived health and well-being, due to the cross-sectional nature of the data. When stratifying the results from the questionnaires on health and well-being by the two identified parental clusters, no notable differences were found, other than slightly higher percentage of women being exposed to active or passive smoking. However, this study shows the variety in combinations of adversities faced by vulnerable women, and can contribute to efforts made to define vulnerability, for research, but also for (social) care practice.

Another limitation of the study is the absence of information about the ethnic background of the women. This information is not regularly collected by the social care providers. Therefore, it was not possible to identify the influence of, for example, racism and stigmatization on the identified problems. However, information on the spoken language was available and can to a certain extent provide information on ethnic background. For a considerable portion of the collected data, we depend on the information collected and registered by the social care professionals. As a result, not all data are as precise or complete as expected from a study setting. On the other hand, the close collaboration with the social care professionals enabled the study team to include and follow such a large number of highly vulnerable pregnant women.

This work presents the first results of the larger Mothers of Rotterdam program, where highly vulnerable pregnant women receive social care and are followed over time for faced adversities and perceived health and well-being. More and longitudinal work will follow from this study, with the current work serving as a solid background, supporting the ideology on which the Mothers of Rotterdam program is founded. The number and complexity of problems the women in this study daily face not only influence their social and economic position in society, but also their health and well-being. On a more subtle scale, living in deprived circumstances is associated with a prolonged exposure to elevated levels of stress, higher prevalence of depressive symptoms and overall worse mental health status compared to their affluent peers [5, 6, 8, 31]. During pregnancy, these unfavourable effects not only affect the woman herself, but also the unborn child. Several indicators of a low socioeconomic position, like living in a deprived neighbourhood, low income or educational attainment, are consistently associated with increased risks of suboptimal growth, preterm birth and perinatal mortality [14, 26, 32, 33]. After birth, the adverse effects of living in deprived circumstances continue to negatively influence the growth and development of the child [34, 35]. The transgenerational effects of low socioeconomic status are not only present in health outcomes, but are also reflected in the social and economic potential of the coming generations.

Taking all these long-lasting effects of low socioeconomic status over (multiple) generations into account, there is a great need for care programs which can intervene in this vicious cycle. The MoR program aims to do this by providing holistic care to highly vulnerable pregnant women covering problems from all domains, including those specific to the (unborn) child. The program uses the pregnancy of these women as window of opportunity for signalling vulnerability, and motivating them to take action. In the Netherlands, almost all women use medical care during their pregnancy or childbirth, and this moment of contact is used to detect potential vulnerability and refer these women to the appropriate (social) care. By improving the cooperation between the medical and social care domains on the level of the care providers [36, 37], this window of opportunity can be optimally used by not only promoting screening for vulnerability, but also providing appropriate care pathways.

With this study, we provide a unique insight into a group of highly vulnerable pregnant women; what adversities they face and how they experience their own health and well-being. Many different adversities and complexity in interplay of these adversities were found, and not only their self-sufficiency was suboptimal, but also their subjective health and well-being was suboptimal. Social care professionals identified problems on multiple life domains that were also considered problematic by the women themselves, although other life domains (including mental health) were regarded differently. More insights into this complex interplay of social adversities and health related problems is needed to optimize care for these highly vulnerable women and their (unborn) children.

## Supplementary Information

**Additional file 1: Supplementary Table 1.** Comparison between participants and non-participants.

## Data Availability

The datasets used and/or analyzed during the current study available from the corresponding author on reasonable request.
