# Supplementary material for: Complexity and interplay of faced adversities and perceived health and well-being in highly vulnerable pregnant women—the Mothers of Rotterdam program
Source: BMC Public Health. 2023 Jan 6;23:43. doi: 10.1186/s12889-023-14975-7 (PMC9817271; doi:10.1186/s12889-023-14975-7)
Supplement: Supplementary file 1 — Additional file 1: Supplementary Table 1. Comparison between participants and non-participants. [file 12889_2023_14975_MOESM1_ESM.docx]

| **Supplementary table 1. Comparison between participants and non-participants** | | | | |
| --- | --- | --- | --- | --- |
|  | | | **Non-participants**  N=57 | **All Participants**  N=862 |
| Maternal age (mean in years, range) | | | 26.2 (17-40) | 27.5 (15-53) |
| Deprived neighbourhood* | | | 38 (66.7) | 547 (63.5) |
| Spoken language | | |  |  |
|  | | Dutch | 50 (87.7) | 742 (86.1) |
|  | | English | 1 (1.8) | 33 (3.8) |
|  | | Arabic | 0 (0.0) | 64 (7.4) |
|  | | Polish | 0 (0.0) | 7 (0.8) |
|  | | Turkish | 0 (0.0) | 2 (0.2) |
|  | | Spanish | 0 (0.0) | 6 (0.7) |
|  | | Other / Unknown | 6 (10.5) | 8 (1.0) |
| Duration of pregnancy  (mean in weeks, range) | | | 17.1 (4-33) | 20.7 (4-40) |
|  | First trimester | | 22 (38.6) | 197 (22.9) |
|  | Second trimester | | 25 (43.9) | 398 (46.2) |
|  | Third trimester | | 10 (17.5) | 267 (30.9) |
| Referring party | | |  |  |
|  | Obstetric professional | | 41 (71.9) | 483 (56.0) |
|  | Social care professional | | 1 (1.8) | 35 (4.1) |
|  | Maternity care | | 1 (1.8) | 35 (4.1) |
|  | Preventive Child Health Centre | | 0 (0.0) | 14 (1.6) |
|  | Application by woman herself | | 3 (5.2) | 106 (12.3) |
|  | Other | | 7 (12.3) | 159 (18.5) |
|  | Unknown | | 4 (7.0) | 30 (3.5) |
| Data are presented as number (N) with percentage (%) unless indicated otherwise. * living in a deprived neighbourhood was based on the residential address of the mother at time of referral [25]. | | | | |
